# Rational management of food intolerance in an elite soccer club

**DOI:** 10.1186/1550-2783-8-S1-P36

**Published:** 2011-11-07

**Authors:** Fabrizio Angelini, Fulvio Marzatico, Gianluca Stesina, Luca Stefanini, Alessandro Bonuccelli, Daniela Buonocore, Sara Rucci, Fabrizio Tencone

**Affiliations:** 1 Society of Sport Nutrition and Wellness, Italy; 2 Medical Staff, Juventus FC, Turin, Italy; 3 Laboratory of Pharmacobiochemistry, Nutrition and Nutriceuticals of Health, University of Pavia, Pavia, Italy

## Background

Gastric intestinal, skeletal muscle and neurological symptoms are just some of the possible effects of alimentary intolerances that may represent a risk for one's health and may frustrate the athlete's practice benefits and thwart the performance. The immunological tolerance recovery and the re-establishment of a normal diet are generally reached by means of strict dietetic schemes (a turnover or elimination diet) requiring a strong effort into changing one's diet habit. In the elite soccer athlete, an intense competitive schedule including long transfers represents another risk to these dietetic therapies fulfilment that may even worsen the symptomatology once the allergens responsible for the intolerances are again within the diet.The purpose of this study was to objectively estimate an food intolerance in elite soccer players who have foodintolerance symptoms, by arranging a nutritional plan for the immunological tolerance recovery, the symptomatology improvement and to test the effectiveness in terms of nutritional situation by means of the BIVA, assuming a close relation among food intolerances, inflammatory reactions and nutritional status.

## Methods

Eight males 32.5 ± 1.9 years old soccer players and BMI 24.9 ± 1.1 (Average ± DS) with symptoms of possible food intolerance (gastralgia, headache, intestinal meteorism, diarrhoea, constipation, nausea) of an Italian Serie A soccer team were subjected to the ALCAT test (IMGeP, Milan, Italy) before and after eight months of a personalized nutritional treatment. The athletes body composition was basally valued and at the end of the BIVA analysis (50 kHz, BIA 101 RJL, Akern Bioresearch, Florence, Italy).

## Results

The athletes tested, with food intolerance symptoms, were ALCAT test variously positive. The personalized nutritional treatment based on moderation rather than on drastic elimination of reactive foods and complying with the specific nutritional needs of the elite soccer player led to a nearly complete resolution of the first symptoms as the clinical evaluation and the post-treatment ALCAT test results demonstrate. Parallel to these results a significant shift of the mean impedance vector was observed (Hotelling T2 test, p < 0.0001), so indicating a more favourable condition of the soft tissues (hydration and/or mass) with no BMI variation (p<0.05).

## Conclusions

The ALCAT test seems to be able to detect the food intolerance reactions when it is applied to patients with initial specific symptoms. A personalized and flexible nutritional therapy based on moderation and rational elimination of reactive foods seems to be working and be suitable for the elite athlete whose specific logistic necessities ( for example long travels) discourage the classic dietary regime. An efficient handling of the food intolerances seems to lead to a nutritional condition improvement, maybe reducing the concerned inflammatory situation as observed in body composition changing, which may influence the sports performance.

